# Advances in 3D neuronal microphysiological systems: towards a functional nervous system on a chip

**DOI:** 10.1007/s11626-020-00532-8

**Published:** 2021-01-12

**Authors:** Wesley A. Anderson, Alexander Bosak, Helena T. Hogberg, Thomas Hartung, Michael J. Moore

**Affiliations:** 1 AxoSim, Inc., New Orleans, LA USA; 2 Department of Biomedical Engineering, Tulane University, New Orleans, LA USA; 3 Center for Alternatives to Animal Testing (CAAT), Johns Hopkins University, Baltimore, MD 21205 USA; 4 CAAT-Europe, University of Konstanz, Konstanz, Germany; 5 Brain Institute, Tulane University, New Orleans, LA USA

**Keywords:** Neuroscience, Microphysiological systems, Nerve-on-a-chip, Brain organoids, 3D culture, Microengineering

## Abstract

Microphysiological systems (MPS) designed to study the complexities of the peripheral and central nervous systems have made marked improvements over the years and have allowed researchers to assess in two and three dimensions the functional interconnectivity of neuronal tissues. The recent generation of brain organoids has further propelled the field into the nascent recapitulation of structural, functional, and effective connectivities which are found within the native human nervous system. Herein, we will review advances in culture methodologies, focused especially on those of human tissues, which seek to bridge the gap from 2D cultures to hierarchical and defined 3D MPS with the end goal of developing a robust nervous system-on-a-chip platform. These advances have far-reaching implications within basic science, pharmaceutical development, and translational medicine disciplines.

## Introduction

Microphysiological systems (MPS) designed to study the complexities of the peripheral and central nervous system have made marked improvements over the years and have allowed researchers to assess in two and three dimensions the functional interconnectivity of neuronal tissues. The recent generation of brain organoids has further propelled the field into the nascent recapitulation of structural, functional and effective connectivity which is found within the native human nervous system. Herein, we will review advances in culture methodologies, focused especially on those of human tissues, which seek to bridge the gap from 2D cultures to hierarchical and defined 3D MPS with the end goal of developing a robust nervous system-on-a-chip platform. Rather than provide a comprehensive review on this extensive subject, we have endeavored to highlight a number of important design considerations that are especially important for developing models of the nervous system. We discuss features including substrate and tissue mechanics, inclusion of neuron and glial subtypes, cell-cell interactions, representative histoarchitecture, functional assessments, and the emerging importance of 3D configuration. We then turn to how those design features are being implemented specifically in models of the central nervous system and of peripheral nerve. We then briefly discuss some translational applications of these models and conclude with a discussion of improvements that are expected in key areas with a nod toward future development. These advances have far-reaching implications within basic science, pharmaceutical development, and translational medicine disciplines.

## Current Challenges in Drug Development

Millions of Americans suffer from neurological disorders such as Alzheimer’s disease, Parkinson’s disease, multiple sclerosis (MS) and amyotrophic lateral sclerosis (ALS) and novel therapeutics are necessary to treat said conditions. Globally, in 2016, neurological disorders were the leading cause of disability-adjusted life years (276 million) and second leading cause of deaths (9 million) (Feigin and Vos 2019). Approximately 15% of children in the US ages 3 to 17 yr were affected by neurodevelopmental disorders (Environmental Protection Agency 2015). Notably, the opioid crisis within the United States of America has resulted in astronomical social and financial burdens with a running total of over 750,000 deaths (Centers for Disease Control 2020) and more than $72.4 billion spent related to opioid use disorder over 15 yr (Leslie *et al*. 2019) spent on care. To both overcome this current crisis and address the broader need for streamlining within the drug development pipeline, more physiologically relevant models are needed to screen potential candidate compounds in nascent testing stages. The overall goal should be to rule out cytotoxic drugs and drugs without human efficacy earlier, thus reducing time and money spent on determining appropriate candidates.

While poor launch probability is pervasive across all medical disciplines, drugs developed targeting nervous system disorders are the least likely to succeed beyond phase I and phase II clinical trials and only cardiovascular disease is ahead for phase III attrition (Dowden and Munro 2019). The failure rate is especially high for neurodegenerative drugs where, e.g., 99.6% of experimental drugs for Alzheimer’s disease have not made it to the market (Pistollato *et al*. 2016; Mohs and Greig 2017). Further, most treatments for central nervous system disorders do not affect the course of the disease but rather serve as palliative therapy. Deeper understandings in neuronal and glial functionality that can be studied using discrete, testable three-dimensional microphysiological systems are imperative to the development of therapeutics that go beyond this limitation of symptom management and move more towards regenerative therapies.

## Neural MPS Tissue Design Considerations

**Figure 1.**
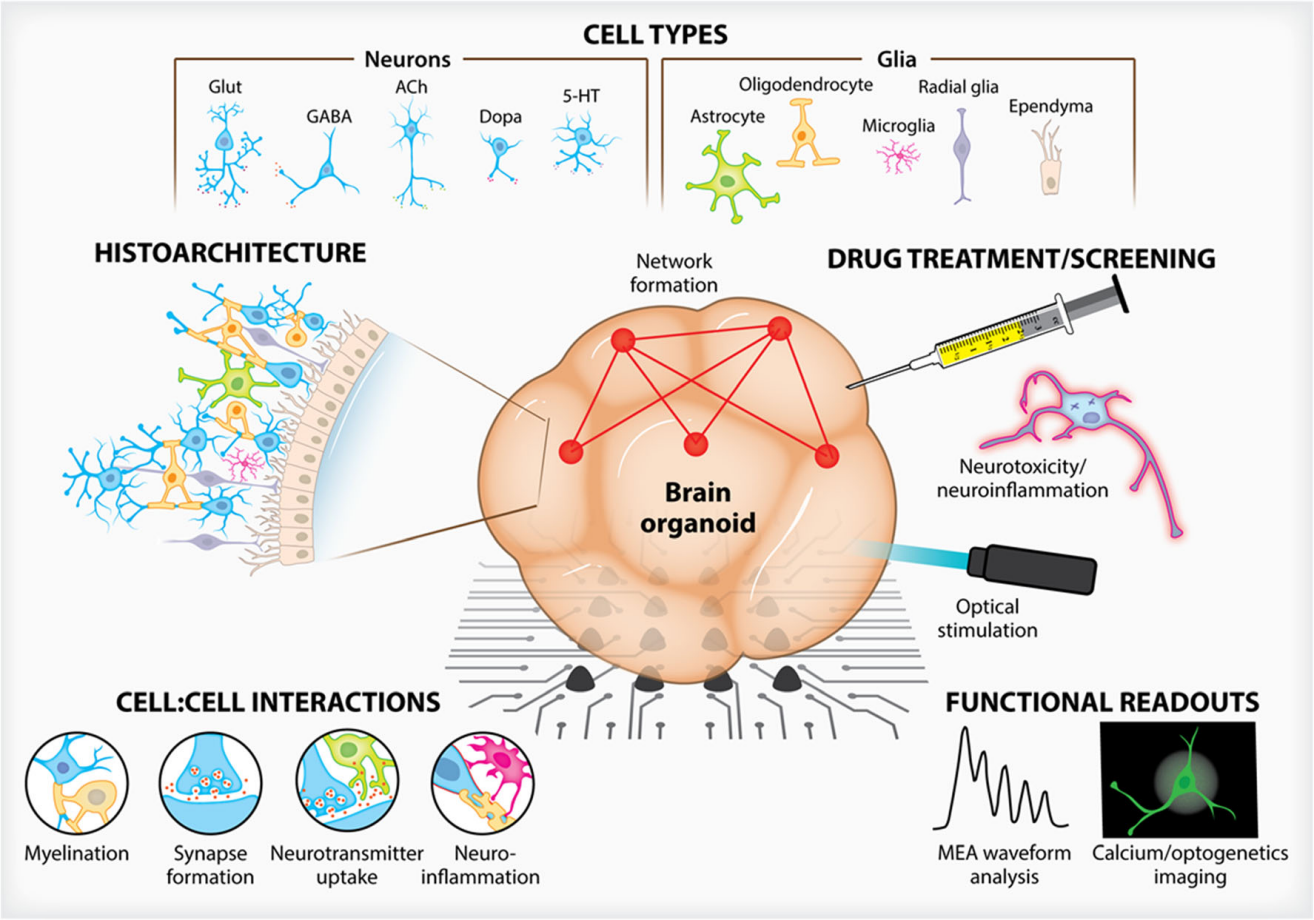
Three-dimensional brain organoid models. Advances in culture methodologies have allowed researchers to build complex, heterogenous brain organoids that are both structurally similar to in vivo architectures and provide meaningful functional readouts. Brain organoids, over traditional two-dimensional cultures, can integrate a myriad of neuronal and glial cell types which exhibit histoarchitecture and cell:cell interactions not possible on planar surfaces. Whether situated atop MEAs or engineered to contain optogenetic constructs, drug screening can be performed to assess brain organoid function and viability.

**Figure 2.**
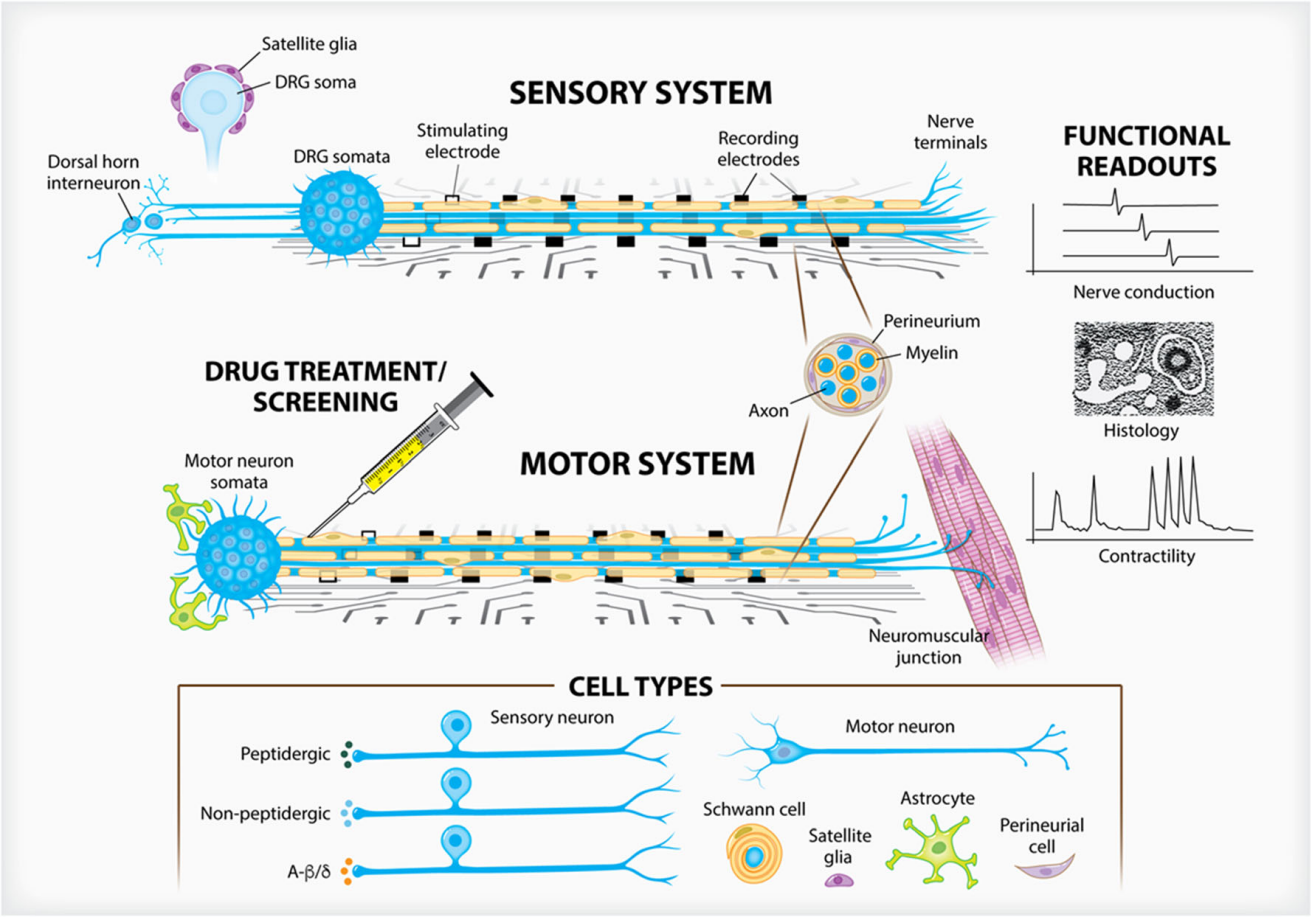
Three-dimensional peripheral nerve models. Peripheral nerve anatomy informs researchers to create 3D constructs that respect the compartmentalized and polar features found naturally. By containing soma either within restrictive chambers or growing them as spheroids, axon and glial outgrowth can be directed along MEA surfaces to assess electrophysiological function. Sensory neural constructs (*top*) can integrate a variety of neural subtypes and be used to study satellite glia in neuropathic pain models and the subsequent input into the dorsal horn. Motor neuron constructs (*bottom*) are currently being integrated into multiorgan NMJ models. Both systems are able to be myelinated by Schwann cells. Nerve fascicle formation, along with perineurial glia, can be studied.

The advent of stem cell technologies and bioengineering in cell culture have led to the development of organotypic cultures, i.e., organoids, organ-on-chip, and human-on-a-chip (multiorgan) approaches, jointly termed microphysiological systems as they replicate some organ architecture and physiology (Figs. 1 and 2). This term (MPS) encompasses the use of various culture and engineering strategies, such as the aforementioned methods, and differs from basic cell culture techniques by seeking to incorporate aspects of the tissue microenvironment to recapitulate some organ-level functionality without requiring in situ or in vivo methods. Their status was summarized in two stakeholder workshops (Marx *et al*. 2016, 2020). Implementing these organotypic cultures within robust microphysiological systems requires the consideration of a multitude of factors that contribute to the success of the system as determined by the physiological relevance and ultimate translatability. Key enabling features to consider include overall structure, individual and population cell:cell interactions, and functional readouts necessary to answer discrete, testable hypotheses. These biomechanical, biochemical, architectural and cell-based cues all contribute to the recapitulation of niches found within the nervous system with each factor working in concert with another. Here we outline examples of advancements made from tuning these key enabling features of MPS designs towards nervous system modeling.

### Substrate and Mechanics

Recreating a physiologically relevant biochemical and biomechanical microenvironment is a key concern for any cell biologist when beginning an *in vitro* study. Extracellular matrix proteins are integral to creating biological niches, which promote the differentiation (Morante-Redolat and Porlan 2019) and maintenance of specific cell types, neurons, and glia in particular. Neural cell cultures have historically and still continue to be grown upon planar polymer and glass surfaces derivatized with extracellular matrix proteins, such as collagen IV, fibronectin, and laminin (Gordon *et al*. 2013). Laminins have proven to be of particular importance to neural development and functionality with laminin-111, the primary component of Matrigel (Hughes *et al*. 2010) and that produced by mouse Engelbreth-Holm-Swarm sarcoma cells, being the most used. While laminin-111 has proven a stalwart substrate, exploration of other isoforms, specifically those with the aphla-5 domain, has shown improved development of network formation (Hyysalo *et al*. 2017) and activity (Hyvarinen *et al*. 2019). Indeed, using a more targeted isoform could be of importance when attempting to recapitulate biological niches which have been shown to contain specific laminins *in vivo* (Nirwane and Yao 2018).

Mechanical characteristics of 2D or 3D platforms influence greatly cell density (Previtera *et al*. 2010), development (Barnes *et al*. 2017), and functionality (Barnes *et al*. 2017) with changes to material moduli helping to achieve either healthy homeostasis or to recapitulate disease environments (Wen *et al*. 2018). Studies have also sought to define critical stiffness parameters with 2D neurite outgrowth on thin films of methacrylate copolymers (Tuft *et al*. 2014), polydimethylsiloxane (Zhang *et al*. 2014), and polyacrylamide (Tanaka *et al*. 2018) consistently showing higher moduli promoting increased neurite outgrowth when grown on planar surfaces. Of note is the supraphysiological moduli employed within some studies that can be orders of magnitude above what is seen *in vivo*. Within physiological gradients, stiffness is still observed to affect network formation and outgrowth atop PDMS substrates (Lantoine *et al*. 2016). Interestingly, neurons continue to exhibit outgrowth on soft substrates (hundreds of Pascals) while astrocyte growth can be restricted (Georges *et al*. 2006). Avoiding the induction of possibly pathological states as seen in stiffer environments found *in vivo* such as a glial scar (Horner and Gage 2000) should therefore be considered when deciding a material’s modulus. Additional to material modulus, restricting embryonic stem cell (ESC)–derived brain organoids between two surfaces has provided some insight into the nascent stages of cortical folding. Controlling the material stiffness, growth compartment and possibly also hydrostatic pressure that is crucial to in vivo development may lead to more fully realized organoids with distinct cortical structures (Karzbrun *et al*. 2018).

### Neuron/Glial Cell (Sub)types

Culturing of neuronal and glial cells has been extensively reviewed prior (Gordon *et al*. 2013; Belle *et al*. 2018). While many of these culture systems focus on non-human cultures, emerging protocols for the rapid generation of human (h) nervous systems from induced pluripotent stem cells (iPSC) is of particular interest to translatable MPS development. Tuning defined media formulations, incorporating inducible promoters and exploring 3D hiPSC differentiation have allowed for cellular differentiation timeline reductions from months to weeks, depending on the cell type. For example, oligodendrocytes can, via a doxycycline-inducible SOX10 promoter, be formed within roughly 20d (Garcia-Leon *et al*. 2018). Strategies requiring no genetic footprint require longer differentiation timescales with the best 3D development strategies taking 30wk (Madhavan *et al*. 2018) to form mature, myelinating oligodendrocytes. Similarly, hiPSCs can be differentiated into astrocytes in cortical spheroids using solely growth factor cocktails and be maintained for a staggering 590d in culture (Sloan *et al*. 2017). Many techniques work towards the end of improving differentiation timelines, especially with tightly defined culture regimens that accelerate differentiation of hiPSCs towards a neuronal progenitor lineage (Walsh *et al*. 2017). Further development of these rapid hiPSC differentiation strategies, especially those which result in co-cultures of neurons and glia, will be required in order to progress scalable MPSs for high-throughput screening applications.

### Cell-Cell Interactions

Beyond characterizing the functionality of neurons, the ability to analyze interactions of neurons to glia is paramount to faithfully modelling *in vivo* processes, especially with the increasing appreciation for glia within neuropathologies such as Parkinson’s (Tremblay *et al*. 2019), Alzheimer’s (Y. S. Kim *et al*. 2018) and ischemic stroke (Hersh and Yang 2018). Powerful tools such as genetically encoded calcium reporters have allowed for the rapid analysis of signaling within astrocyte populations in response to neuronal activity (Savtchouk *et al*. 2018). Apart from rapid signaling, characterizing the metabolic outputs resulting from neuron:astrocyte interaction has been studied in 3D substrates, specifically the monitoring of glucose and acetate metabolism (Simao *et al*. 2016).

As oligodendrocytes appear to show preferential myelination of certain neuronal subtypes *in vivo* (Zonouzi *et al*. 2019), creating 3D models to study their cell-cell interactions with neurons is vital to rapidly assess development and response to injury. Furthermore, myelination is a key process for neuronal functionality and several human neurologic diseases have been associated with dysfunctional oligodendrocytes and myelin deficits, such as schizophrenia, multiple sclerosis (MS), amyotrophic lateral sclerosis (ALS) and periventricular leukomalacia (PVL) (Noseworthy *et al*. 2000; Billiards *et al*. 2008; Kang *et al*. 2013; Najjar and Pearlman 2015). Therefore, this feature becomes particularly important to include in MPS for drug development. However, oligodendrocyte differentiation in 3D has been assessed with exceptionally long *in vitro* culture times (up to 235d (Marton *et al*. 2019)), which may not be compatible with drug development strategies and timelines. This study was however able to show functional myelination and electrophysiological readouts sensitive to drugs blocking ionotropic glutamate receptors expressed by oligodendrocytes. Further, these oligodendrocyte spheroid cultures contained astrocytes and neurons and were able to track individual cell migration and mapped morphology and maturation via live imaging. Conversely, in a truly reductionist manner, models have been created which remove the neuron itself and opt for artificial axons to study oligodendrocyte myelination on 3D scaffolds (Espinosa-Hoyos *et al*. 2018), with ensheathment being promoted in laminin- and stiffness-dependent manners. Tuning cell-surface markers to help model myelination of specific neuron subclasses may even pave the way for further defining oligodendrocyte heterogeneity and subclasses, an underappreciated characteristic of a “functionally homogeneous” cell type (Marques *et al*. 2016).

Non-neuronal cells that serve barrier functions have been of interest when creating MPSs. The blood-brain barrier (BBB) is widely the most studied barrier and has been reviewed extensively prior (Banks 2016; Villabona-Rueda *et al*. 2019). 3D *in vitro* models of the BBB have been built to study Alzheimer’s disease (Shin *et al*. 2019), interactions between astrocytes and vascular cells (Campisi *et al*. 2018), and the modelling of metastatic brain tumors (Xu *et al*. 2016). Organoid models have even been created which produce cerebrospinal fluid from choroid plexus epithelia derived from human stem cells (Pellegrini *et al*. 2020). Notably, these choroid plexus organoids contained pertinent transporters integral to barrier functionality and also secreted a host of relevant proteins one would expect to find in cerebral spinal fluid (49/50 of the most detected proteins in the organoid model are also found within *in vivo* samples). And while not necessarily a barrier-forming cell, satellite glia within the periphery envelope dorsal root ganglion somata and perform a litany of functions related to pain processing (Costa and Moreira Neto 2015), sympathetic transmission and neuron survival (Enes *et al*. 2020). There is also evidence to suggest that satellite glia serve as a reservoir of arrested Schwann cells that, via regulation of cadherin-19, may leave the soma and become mature, myelinating cells should cues arise, such as in injury (George *et al*. 2018).

### Histoarchitecture

Modeling any tissue with fidelity ultimately leads to consideration of 3D microarchitecture, as all tissues are inherently 3D. The aforementioned characteristics culminate to recapitulate niches found in neural tissue with the now added dimensionality of non-planar culture surfaces. For example, while the fasciculation or bundling of nerves is of importance to modeling the peripheral nerve, this would be an erroneous design criterion for the central nervous system where neuroanatomy adopts stratified architectures in regions such as the cortex or hippocampus. While organotypic slice cultures are still used to study the spinal cord (Musto *et al*. 2019) and brain, recent advances have seen central nervous system neurons grown on mechanically controlled anisotropic scaffolds (Kim *et al*. 2017), porous microlattices (S. Li *et al*. 2018) and 3D bioprinted substrates (Antill-O’Brien *et al*. 2019) in order to recreate aspects of 3D macrostructure or neural connectivity seen in the brain. The peripheral nerve has also seen advancements in growing explant or spheroid cultures within anisotropic materials (Anderson *et al*. 2018; George *et al*. 2019) and growth restrictive hydrogels (Huval *et al*. 2015; Nguyen *et al*. 2019; Sharma *et al*. 2019; Kramer *et al*. 2020), respectively. Ultimately, the inherent qualities of anatomical histoarchitecture should guide the design of microarchitectural features of microphysiological devices.

### Functional Assessment

Functionality of a neuronal system is paramount for any meaningful data acquisition. Electrophysiology outcomes such as those acquired via microelectrode arrays (MEA) are the emerging gold standard for *in vitro* studies and multiple studies have highlighted the difference between 2D and 3D systems. Stiff, laminin-coated glass beads used as a 3D network positioned above an MEA showed variant burst rates and stimulus-evoked responses compared with 2D culture of e18 rat hippocampal neurons (Frega *et al*. 2014). Softer substrates, such as anisotropic alginate-based scaffolds (Anderson *et al*. 2018; George *et al*. 2019) or carbon nanotube-containing PDMS (Bosi *et al*. 2015), have also shown varied activity including capabilities of delineating capsaicin-dependent spikes of peripheral nerve culture and improving overall hippocampal neuron activity, respectively. With 3D systems outperforming 2D, future development should surround exploring further how 3D systems may affect arborization (Kayal *et al*. 2019), synaptic maturation and functional activity, especially in the presence of drugs.

Microscopy utilizing ratiometric fluorescent dyes, genetically encoded calcium reporters and two-photon imaging techniques has allowed for the real-time assessment of synchronous activity in many tissue types, including neurons (Grienberger and Konnerth 2012; Mitani and Komiyama 2018). Similarly, the utilization of optogenetic technology has been used in 3D neuronal models to induce action potentials selectively via specific genetic promoters and to activate organ systems through synapses (Renault *et al*. 2015; Lee *et al*. 2019), such as the neuromuscular junction (Osaki *et al*. 2020). Multiplanar imaging of complex neural circuits has also improved and allows for the study of neurons both *in vitro* and *in vivo* (Yang *et al*. 2016). Construct dimensionality influences this synchronous behavior as seen with hippocampal neurons grown upon planar graphene surfaces or embedded within 3D graphene foams (Ulloa Severino *et al*. 2016). By affecting the structural connectivity of the tissue (i.e., culturing with the foam), the functional connectivity was improved, resulting in a greater degree of high synchronous activity as compared to only moderate synchronous activity within the 2D controls.

### Emerging Superiority of 3D Neural Cultures

Advancements in 3D culture techniques have presented researchers with two paradigms by which to approach experimental designs. While 2D cell culture configurations will continue to be useful for answering certain questions, it is becoming apparent that 3D culture configurations can best capture the complexity of the nervous system, whether considered by structural, functional, or effective connectivity, to provide the most clinically translatable outcomes. Properly replicating *in vivo* responses cannot be achieved in 2D environments as they do not provide necessary cell signaling cues for normal nervous system development (Alepee *et al*. 2014). Therefore, successfully generating these neuronal connections in vitro requires guided 3D tissue development and maturation (Lai *et al*. 2012) to best recapitulate what is observed *in vivo*. Improved cell:cell interactions and signaling lead to complex structure formation such as the 4-part synapse including the presynaptic and post-synaptic neurons, the astrocyte, and the microglia (Schafer *et al*. 2013). It has been shown that the activity better mimics what is seen *in vivo* when cells are grown in a 3D environment. Cells in the brain develop in a 3D space, and these multicellular complex networks that are formed are important for greater neural function (Frega *et al*. 2014). Comparable networks can have drastically different effects on network bursting and synchrony when cultured in 3D, which may further reveal cellular responses that further mimic *in vivo* responses.

## Modelling the Central Nervous System

The human central nervous system is a complex network of various neuronal, glial and immune cells, which can be divided into discrete architectural areas that define motor, sensory, memory or other functions. By this classical approach, the brain can be assessed by a structural connectivity approach wherein defined areas can be classified neuroanatomically via long-standing neuroimaging techniques. Further characterizing these structures based upon functional readouts (e.g., electrophysiology, fMRI, PET) can show functional connectivity wherein “an observable phenomenon… can be quantified with measures of statistical dependencies” (Friston 2011). This allows for observations of connectivity between brain centers not dependent upon specific experimental models or hypothesis. Experimental approaches lend themselves to what is termed effective connectivity which “corresponds to the parameter of a model that tries to explain observed dependencies” (Friston 2011). With these definitions in mind, the approach to building meaningful platforms to study the CNS should create a functionally connected network of tissue which can be assessed for effective connectivity through experimentation and physiologically relevant outcomes (Fig. 1). As individual *in vitro* studies will undoubtably require various functional outcomes, the desired structural parameters of the chosen model will be largely dependent on the specific questions being asked.

### Microengineered Culture Systems

**Figure 3.**
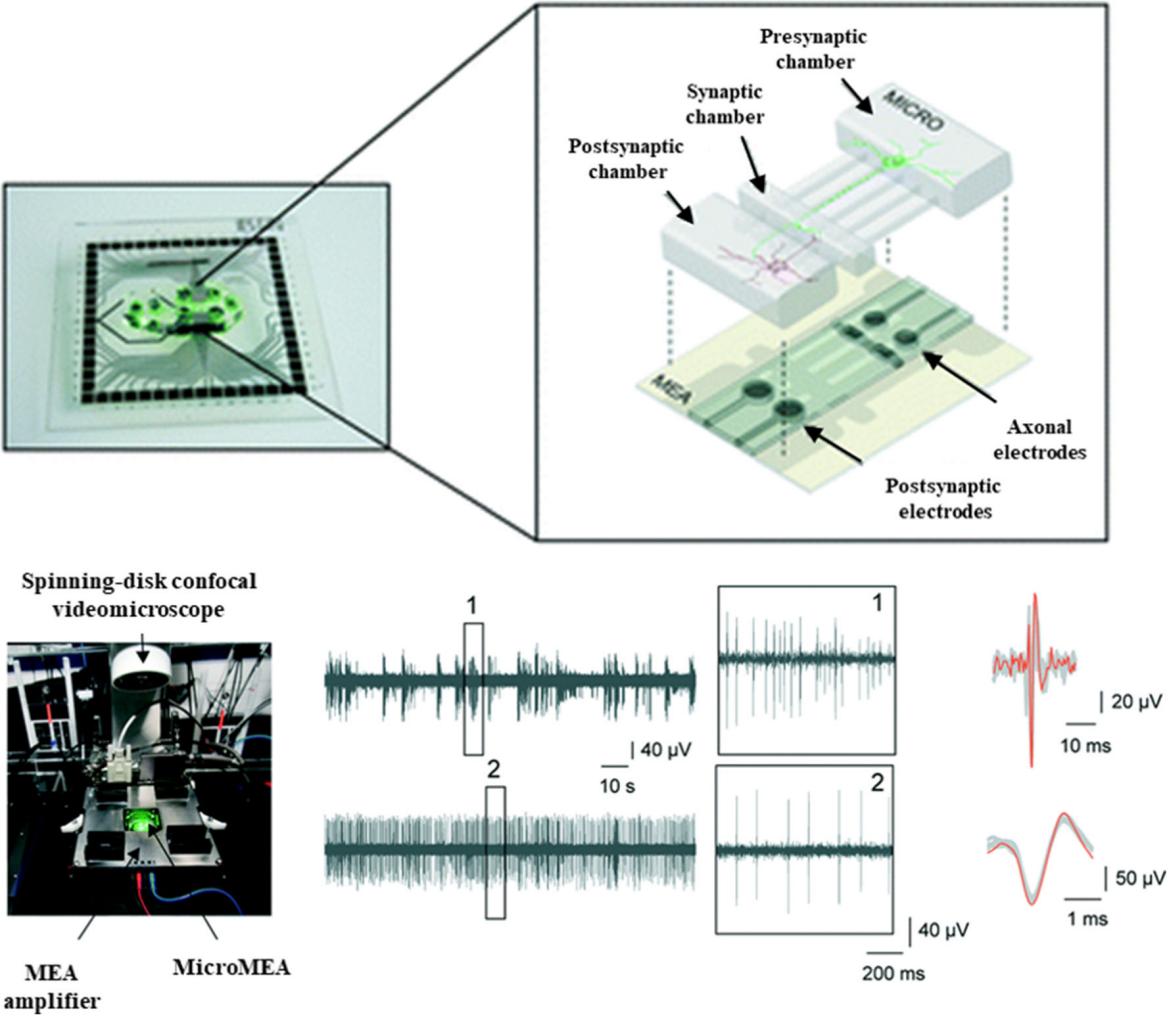
MPS for modeling CNS interconnected functionality has been achieved with engineered culture systems using microfabricated chambers for specifying synaptic connectivity and microelectrodes for evaluating function. Adapted from Moutaux *et al*. (2018), with permission from The Royal Society of Chemistry.

Efforts to use microfabrication techniques to control the organization and connectivity of CNS cells in vitro date back at least several decades to the invention of the eponymous Campenot chamber by Robert Campenot (1977) (Campenot 1977). These culture systems typically make use of relatively simple structural features such as microtunnels and microfluidic channels to organize cells, promote specified connectivity, and enable compartmentalization, often combined with microelectrode arrays for physiological assessment (Fig. 3). Advancements in approaches such as these have been reviewed in detail recently (Nikolakopoulou *et al*. 2020). While typically restricted to 2D formats, these model systems excel especially when it is important to compartmentalize cell types or segregate axons from somas so that drugs or neuromodulators may be applied to one compartment at a time.

### Brain Organoid Models

**Figure 4.**
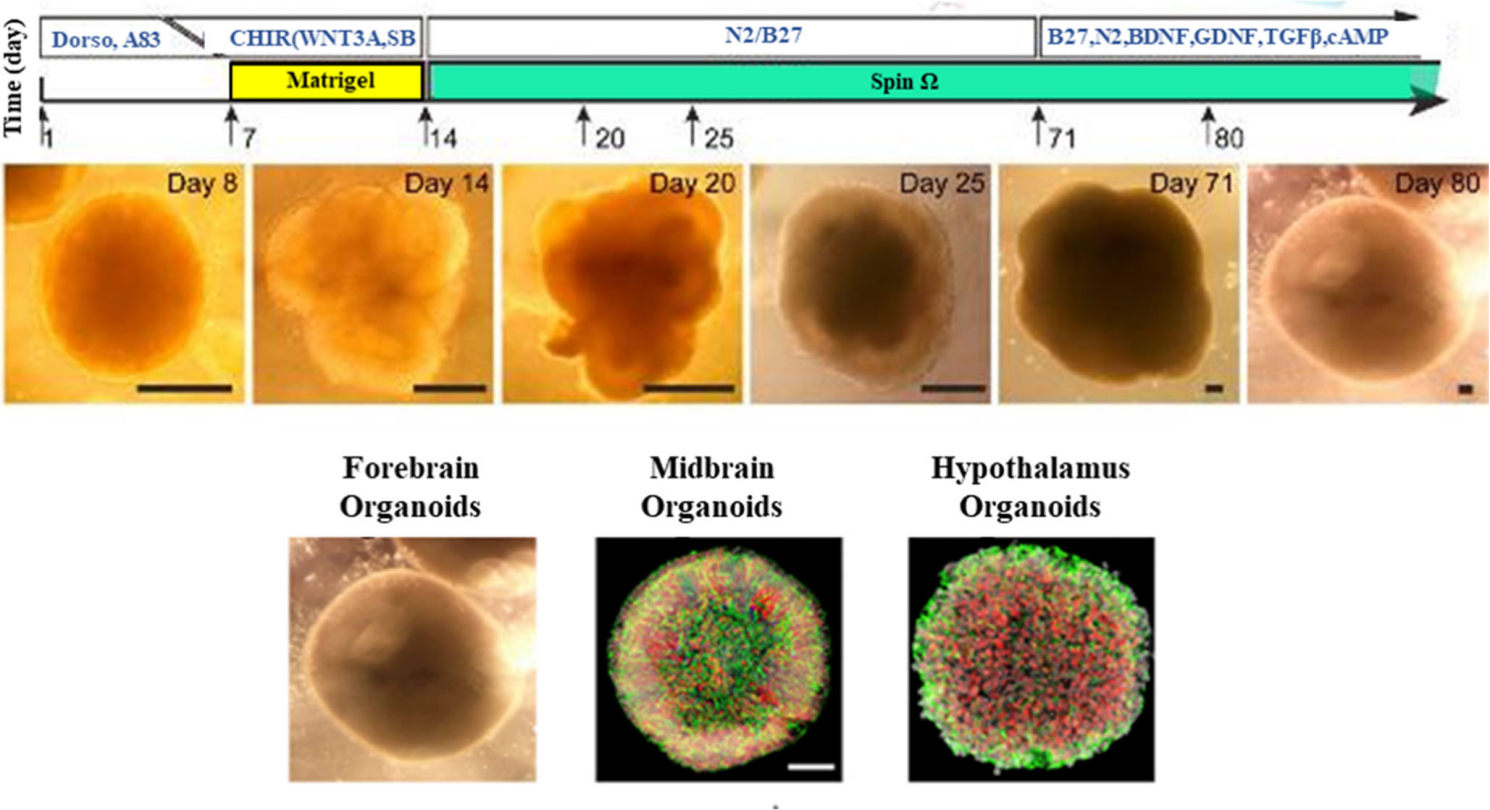
Brain organoids rely on self-assembly during differentiation from pluripotent cells. Adjusting culture conditions has produced large organoids exhibiting self-organized internal structure as well as organoids representing distinct brain regions. Adapted from Qian *et al*. (2016), with permission from Elsevier.

**Figure 5.**
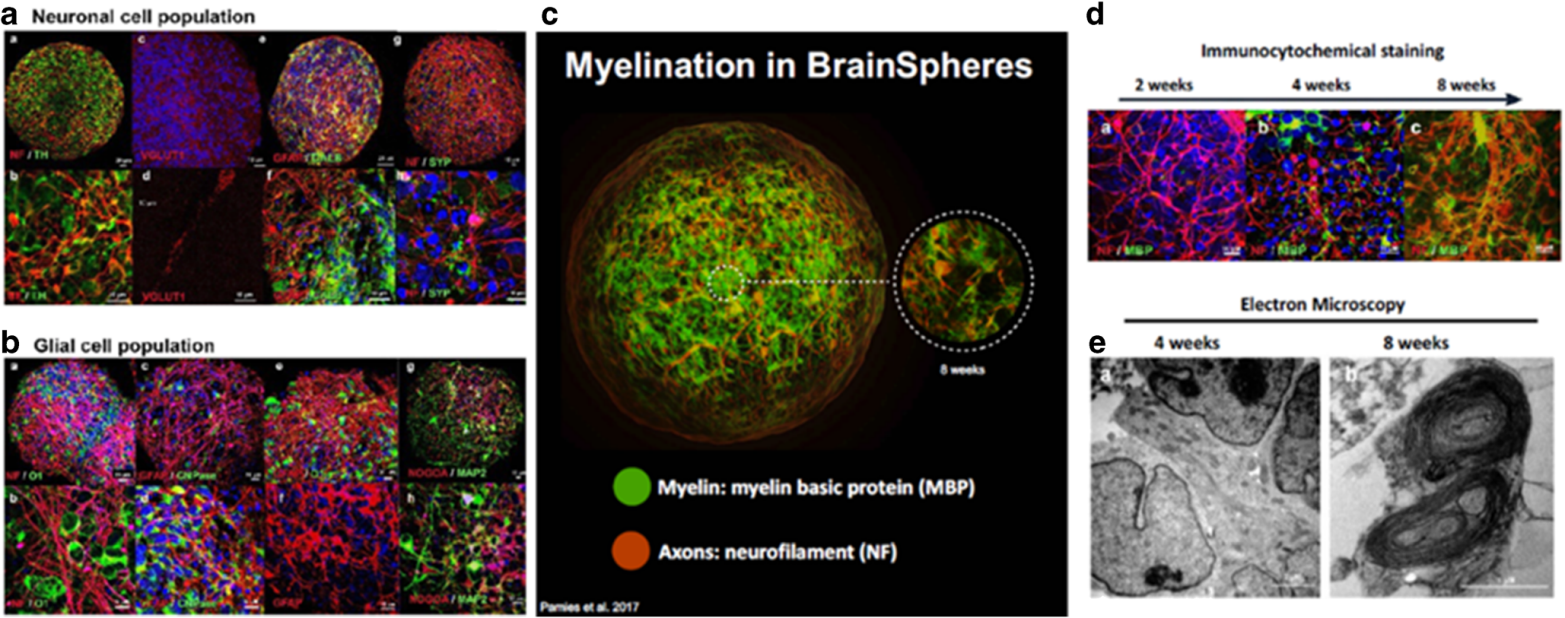
Brain spheroids for the 3D modeling of the central nervous system. Developed spheroids show a diverse population of both (*a*) neuronal and (*b*) glial cells throughout the bulk of the organoid. (*c*) Myelination is also readily apparent and the progression of myelination within the spheroid can be assessed via both (*d*) immunohistochemistry and (*e*) TEM. Reproduced from Pamies *et al*. (2017), under Creative Commons license.

Brain organoids are made by differentiating hiPSCs or embryonic stem cells through a neural lineage to generate the major cells that make up the human brain (Lancaster *et al*. 2013). These models generally include relevant mature cell types of neurons are capable of self-assembly into organoids from distinct brain regions (Fig. 4). Diversity in incorporated cell types and culture conditions yield varying, complex organoids that are capable of being engineered to enable specific neuron:glia compositions and interactions, namely myelination. (Fig. 5). The major neuronal subtypes studied include glutamatergic, GABAergic, dopaminergic and serotonergic neurons. Importantly, cell numbers can be analyzed to study the effect of specific drugs on each cell population and the effect that other cells in the system may be having on each other. Normal development of these cells gives rise to important microstructures of the brain including neuronal axons and dendrites. In this way, synaptic communication can be studied and between excitatory and inhibitory neurons, leading to a deeper understanding of brain circuitry.

These models benefit by allowing for the physiological study of neuronal electrical activity. By plating these on MEA plates, the spontaneous electrical activity can be measured in a high-throughput format. Electrical characteristics can be measured when cells are exposed to chemicals or even exogenous electrical stimuli. The multielectrode arrays also allow for the study of network activity, which is related to more complex cellular interactions. One study has demonstrated the detection of complex oscillatory waves from cortical organoids maturing for long periods in culture. Remarkably, when followed for several months, dynamic oscillatory waves began to give rise to network synchrony that exhibited phase-amplitude coupling (Trujillo *et al*. 2019). Such advanced functional metrics as these may serve to model complex neurological phenomena in vitro that may have implications in disorders such as epilepsy, autism and mental illness.

### Brain Spheroid Myelination

Oligodendrocyte differentiation leads to myelination in brain spheroid models, but many models currently being used do not include this cell type. Oligodendrocyte differentiation from iPSCs has been difficult and only a few organoid models currently include these cell types. One study demonstrated the formation of brain spheroids that include oligodendrocytes and endogenous myelination (Pamies *et al*. 2017) (Fig. 5*c*–*e*). Co-localization of neuronal neurofilament-heavy protein (NF-H) with MBP immunostaining suggested evidence of myelin formation as early as 4wk into differentiation and up to 40% myelinated axons by 8wk of development. Transmission electron microscopy confirmed the presence of compact myelin microstructure by 8wk of development and enabled measurement of G ratios, which may serve as a metric for modeling myelin diseases. This approach may lend itself for use in studying demyelination and remyelination. Cuprizone and lysophosphatidylcholine (LPC) treatment (Vereyken *et al*. 2009) is commonly used to induce demyelination in the CNS in animal models (Zhan *et al*. 2020). With this drop in myelination, this system can also be used for remyelination assays, which would be especially important for studying relapse-remitting multiple sclerosis. The causes for remyelination failure in more progressive phases of multiple sclerosis are unknown (Charles *et al*. 2002) and shows that this could be a powerful tool for these studies.

## Modeling Peripheral Nerve

The peripheral nervous system serves to relay central outputs to target tissues and as the sensory input responsible for gathering normal and noxious stimuli from the extremities and transmitting it to the dorsal horn of the spinal cord. The development of in vitro models of peripheral nerve has been reviewed in detail recently (Pollard *et al*. 2019). Given the unique pseudo unipolar nature of neurons within the dorsal root ganglia, the collection of somas stemming from the dorsal ramus, creating MPSs which are able to recapitulate the distinct anatomical confinement of peripheral nerves is preeminent to design consideration (Fig. 2). Herein, we will discuss these important structural elements, the ability to integrate them with microelectrode platforms and how to approach greater physiological relevance by inducing myelination and assessing drug cytotoxicity.

### Peripheral Nerve Histoarchitecture

**Figure 6.**
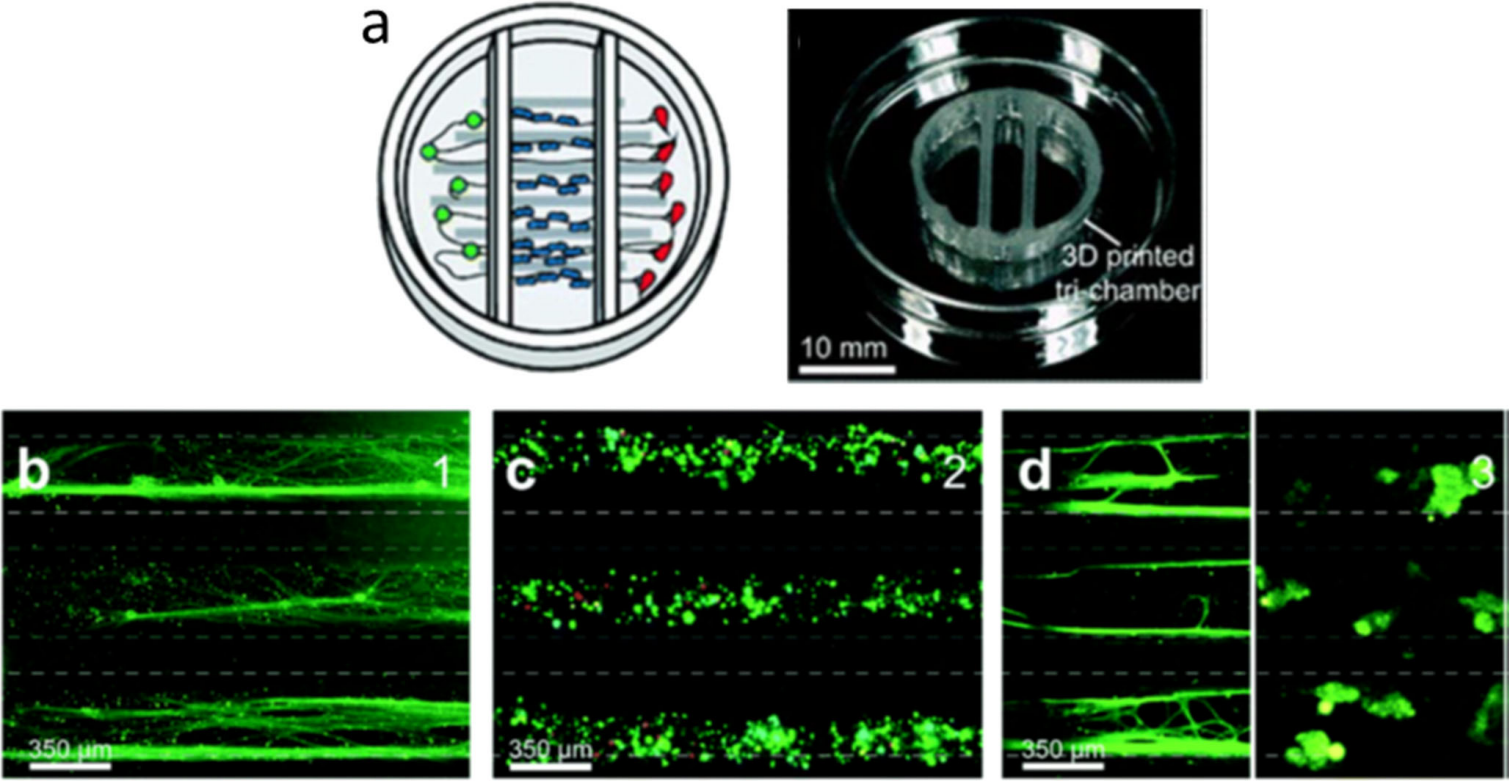
Peripheral nervous system compartmentalization through 3D printed constructs. (*a*) Schematic and formed 3D printed chamber for compartmentalized cell growth. From *left* to *right*, these three chambers contain (*b*) neuron cell bodies, (*c*) Schwann cells growing along axonal projections, and (*d*) axon terminals. Models such as this one can ensure compartmentalized growth and can realize aligned growth of various cell types. Adapted from Johnson *et al*. (2016), with permission from The Royal Society of Chemistry.

As noted above, the peripheral nervous system is composed of neurons which have either a unipolar (motor) morphology with a single axon extending from the soma in the spinal cord to the periphery or else a pseudo-bipolar (sensory) morphology that branches towards the periphery and the dorsal horn of the spinal cord. Merely plating peripheral neurons on planar surfaces does not allow for recreation of this morphology; therefore, a myriad of engineering techniques have been employed to generate MPSs that can appropriately induce polarized and parallel-aligned axon growth mimicking nerve structure. 3D printed microchannels have been able to guide DRG axon outgrowth and co-culture with Schwann cells has shown successful induction of myelination and Schwann cell proliferation (Johnson *et al*. 2016; Sakai *et al*. 2017) (Fig. 6). A rudimentary perineurium has also been achieved within alginate constructs with open capillaries. Coating of these constructs with laminin allowed for nerve outgrowth and enough of a polarizing signal to form perineurial cells marked by tight junction expression and distinct localization around the nerve bundle (Anderson *et al*. 2018).

### MEA Recording

**Figure 7.**
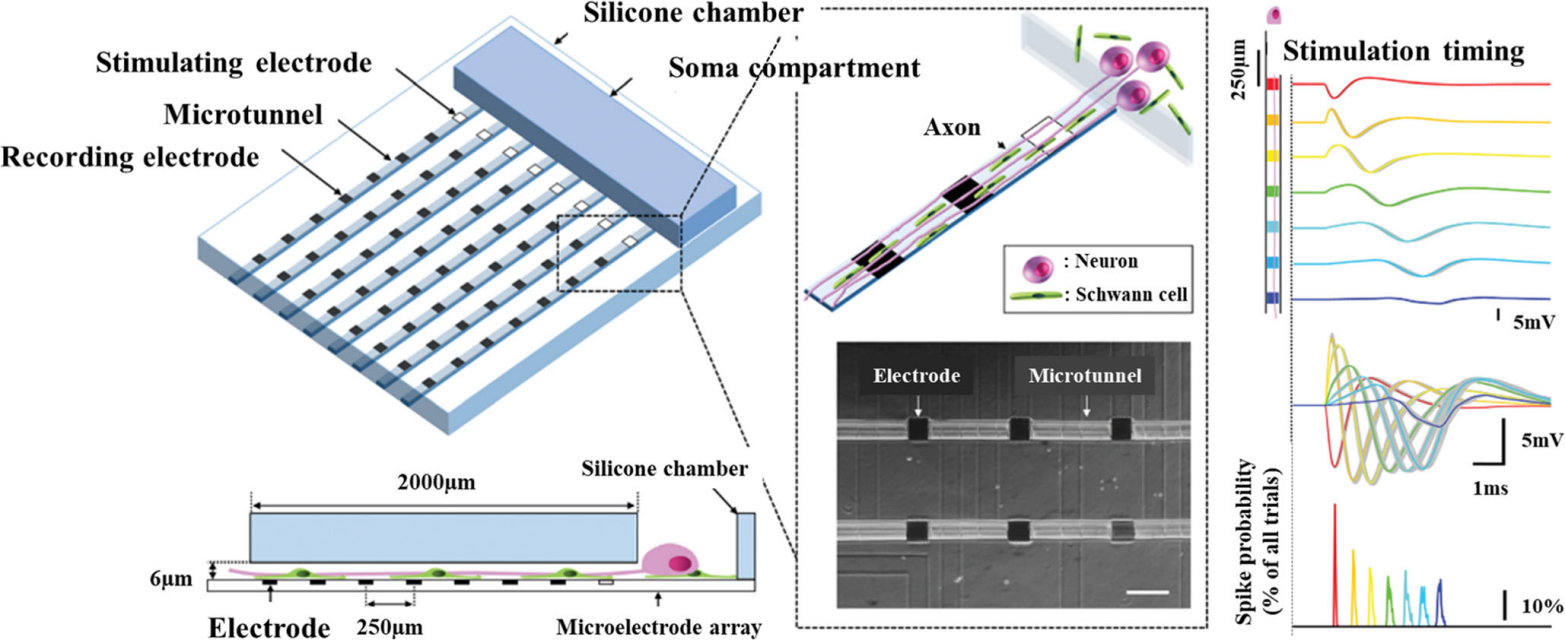
Peripheral nerve MPS demonstrating compartmentalization, polarization, and alignment in 2D, along with integration of microelectrodes for spike sorting and observation of axonal conduction. Adapted from Sakai *et al*. (2017), by permission of Oxford University Press.

*In vitro* recordings of neurons on the microscale have long been accomplished using patch clamp. However, this method allows only for single cell analysis, which does not serve to capture the organ level activity created by 3D MPSs. Electro-optical methods such as channel rhodopsin and calcium signaling imaging such as genetically encoded fluorescent reporters have helped push recordings towards multicell systems, but the microelectrode array remains the gold standard in local field recordings of neural tissue constructs. This technology reviewed by Obien *et al*. (2014) combines tissue on a chip engineering designs with real-time electrical outputs necessary to quantifying neural processes. Of special interest is combining developed 3D culture systems with these traditionally planar recording modalities; progress has been made previously in 2D to this end and has been achieved with compartmentalization, polarization, and tissue alignment (Fig. 7). Bridging this gap would allow for the real-time recording of peripheral nerve organoid cultures over long experimental time courses instead of using manually inserted field electrodes which can damage tissue and introduce contamination.

### 3D Peripheral Nerve MPS

**Figure 8.**
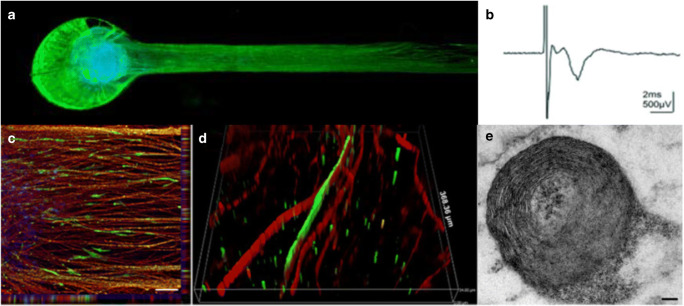
Peripheral nerve modeling and myelination. (*a*) Rat primary sensory neuron tissue grown as a spheroid within a growth-restrictive hydrogel can yield (*b*) induced compound action potentials detected by extracellular field recording. (*c*, *d*) Similar spheroids composed of iPSC-derived human neurons can show outgrowth and myelination that can be confirmed by (*e*) TEM micrographs showing compact myelin rings. Reproduced from Sharma *et al*. (2019), under Creative Commons license.

Peripheral nerve tissue-on-a-chip platforms should build upon microfabrication techniques and tune them towards the development of testable 3D models with physiologically relevant outcomes, notably electrophysiology. These constructs should be able to direct axonal growth, support glial (e.g., myelination) and neuronal (e.g., synaptic transmission) cell functions and be able to record electrophysiological signals with high signal-to-noise ratios. As within any cellular system, maintaining high viability is crucial. Thick tissue sections and large organoids lack vasculature and therefore do not have the ability to supply oxygen deep into tissue. To overcome the formation of necrotic cores, it is then important to maintain a tissue volume that does not exceed a radius or depth of roughly 200μm (i.e., exceeding the passive diffusion distance of oxygen within tissue (Rouwkema *et al*. 2010)). One solution to this problem is the formation of defined spheroids with tunable sizes based upon cell numbers. These spheroids, which exhibit excellent viability (Fig. 8*a*), are able to be manually manipulated and placed within tissue constructs composed of various substrates, such as methacrylated gelatins or more ubiquitous ECM scaffolds such as Matrigel. Notably, these spheroids can be cultured for many weeks without losing functionality. These long-term culture conditions allow for extensive neurite outgrowth in all dimensions with growth-restrictive hydrogels being able to confine dense axon beds to specific areas for electrophysiological recordings. These constructs can then be used for electrophysiological assessment and exhibit peaks that are able to be defined by treatment with drugs (Kramer *et al*. 2020). Additional to neuron function, glia are able to grow and myelinate, more on that next.

### Induction of Myelination

Primary embryonic rat tissue has long served as the basis for peripheral nerve studies and has elucidated mechanisms of myelination by Schwann cells dating back to the 1980s (Eldridge *et al*. 1987). However, myelination of human axons by human Schwann cells in vitro has been much more elusive (Monje 2020). These myelination studies have long existed within 2D systems such as those described above wherein cells are cultured atop ECM coated glass coverslips without any discrete compartmentalization. While some microfluidic chambers and 3D biomaterial constructs have incrementally moved away from this by promoting alignment via construct architecture, generating a dense, aligned 3D nerve with myelination has only recently been achieved with human cells (Sharma *et al*. 2019). Growth restrictive 3D hydrogels have shown capable in generating myelinated human MPSs by combining commercially available motor neuron cultures with Schwann cells. Addition of ascorbic acid to optimized co-seeding densities was able to induce myelination as observed via immunofluorescence and TEM (Fig. 8*c*–*e*). Compound action potentials were able to be recorded with conduction velocity and G ratios able to be quantified. Further development of this system is geared towards the fabrication of peripheral sensory neuronal systems with the promotion of Schwann cell migration being a critical goal to enable myelination of greater length scales.

## Translational Aspects of MPS: Modelling Neuropathies

Given the aforementioned structural and functional benefits of modelling the development and physiology of neural tissues in 3D, it would be reasonable to conclude that studying pathologies within 3D systems is beneficial over models that utilize planar substrates. Organoids in particular provide robust environments to assess both peripheral and central nervous system diseases. The effects of cell proliferation, migration, genetic anomalies, and environmental factors on organoid development and function have been previously reviewed in the context of neuropsychiatric disorders (Amin and Pasca 2018). This has pushed the field of neuroscience and ultimately patient care forward by allowing for basic science approaches to future pathophysiology studies and drug development that do not rely on either post-mortem assessment of tissue or non-invasive techniques (such as MRI, PET, CT) that lack the resolution required to observe ultra-fine structures (e.g., dendritic spines). Here, we will highlight specific areas what have seen advancements of bridging the development of MPS to the translational study of neuropathies, i.e., moving from microphysiological to micropathophysiological systems.

### Connection to Target Organs: Modelling the Neuromuscular Junction

Peripheral nerve models on their own (i.e., motor or sensory neurons individually or in combination with glia) hold the capacity to elucidate many basic science inquiries and are entering nascent stages of allowing for higher throughput capacity for drug screening. By integrating these neural MPSs with the target tissue, their innervation of or providing reception for *in vivo* tissues opens up new avenues of research focusing on pathologies that affect both the peripheral nervous system and said target tissues. The most widely studied target organ structure to-date remains the neuromuscular junction (Omar *et al*. 2020) (NMJ), primarily that of skeletal muscle to study diseases such as Amyotrophic Lateral Sclerosis (ALS), myasthenia gravis (Faustino Martins *et al*. 2020) and Duchenne muscular dystrophy (Afshar Bakooshli et al. 2019). While advanced 2D culture chambers are still being utilized to screen drugs *in vitro* (Santhanam *et al*. 2018), research has begun turning to 3D co-cultures of spinal neurons and skeletal muscles fibers, whether in organoid formats encapsulated within fibrinogen (Afshar Bakooshli *et al*. 2019) or silk fibroin hydrogels (Dixon *et al*. 2018). Importantly, these systems showed functional NMJ formation, measured skeletal fiber contractility, modeled axonal transport of pathophysiological TAR DNA binding protein 43 (TDP-43) in the case of ALS and could even induce a model of myasthenia gravis with patient-derived antibodies targeting NMJs. Innervation of muscular fibers was also able to be sped up in 3D versus 2D cultures as well as more robust fiber formation as defined by contractility, hypertrophy, calcium handling and expression of key receptors, notably the acetylcholine receptor.

### Brain Spheroid Neurotoxicity Assay

Brain spheroids have been shown to be effective models for studying neurotoxicity. Treatment with rotenone (Nzou *et al*. 2018) has led to evidence of neuronal toxicity *in vitro* (Pamies *et al*. 2018). This toxicity was higher in spheroids that were treated earlier in development when compared with spheroids treated at later time points. Dopaminergic neurons were especially sensitive to rotenone treatment and showed selective toxicity at “non-cytotoxic” concentrations, sparing astrocytes and other neuron subtypes. Moreover, the model identified the antidepressant paroxetine as a developmental neurotoxicant where synaptogenesis, neurite outgrowth and the number of oligodendrocyte were significantly decreased at therapeutic blood concentrations (Zhong *et al*. 2020). This demonstrates that this model’s usefulness for studying neurotoxicity (Schmidt *et al*. 2017) and developmental neurotoxicity (Smirnova *et al*. 2014). The use of iPSC also allows for assessment of gene-environmental interactions as donors with different genetic backgrounds can be used.

### Neuroinflammation of the Central Nervous System

The brain spheroid models discussed so far have been composed of cells that arise from the neuroectoderm that make up the CNS, but do not include microglia, another vital cell found in the CNS for normal brain development and function. Microglia are the resident phagocytic immune cells in the brain and spinal cord.

By adding microglia to these spheroid models, neuroinflammation can be studied (Abreu *et al*. 2018). LPS treatment is well documented to cause neuroinflammation and neurotoxicity and is a known stimulator of microglia. Treatment of brain spheroids containing microglia have shown similar responses to what is seen i*n vivo*. Treatment with LPS causes an increase in the expression of inflammatory cytokines. An inflammatory response was also detected in response to flavivirus infection shown in the experiment. This demonstrates the ability of this model to be used in further research of dengue and Zika viruses and how the brain spheroid model also does well for studying neuroinflammation. Finally, the relevance of studying viral infection, replication and spread within the nervous system cannot be diminished, especially due to the recent SARS-CoV-2 pandemic. Brain spheroid models have helped elucidate these key facets of viral tropism within the CNS, and the continued study *in vitro* will help to model the currently unknown long-term effects of this increasingly ubiquitous pathogen (Bullen *et al*. 2020).

### Cancer

Brain MPS have also enabled cancer studies by grafting or inducing for example glioblastoma in brain organotypic cultures. Glioblastoma, one of the most aggressive brain tumors, was introduced into the 3D human stem cell–derived models via genetic editing or by co-culturing with glioblastoma cells (Nayernia *et al*. 2013; Bian *et al*. 2018; Ogawa *et al*. 2018; Linkous *et al*. 2019; Plummer *et al*. 2019; Krieger *et al*. 2020). Such cancer-bearing organoids promise personalized medicine by exploring most effective therapeutics for a given patient as well as the development of novel therapeutics (Plummer *et al*. 2019). Culturing the cancer in a physiological environment and not just as cancer organoids promises to recreate the cancer microenvironment, which is critically impacting on tumor behavior. The approach also allows to directly compare the drug effects on cancer and healthy tissue, defining therapeutic windows.

## Conclusion and Future Perspectives

Microphysiological systems combine facets of bioengineering, molecular biology, material science and computer modeling to best recapitulate natural processes in defined, testable systems. Neural MPSs, for both the peripheral and central nervous systems, have allowed for basic science research in neuroscience to be propelled beyond traditional 2D culture and the integration of 3D culture methods has only furthered neural MPSs. While much progress has been made, opportunities remain in ways to best recreate cytoarchitectures and organ-level anatomy, all while focusing on translatable outcomes such as overcoming challenges in the drug discovery pipeline. Further incorporation of glia, especially satellite glia in the peripheral and microglia in the central nervous system, will expand cell:cell interaction studies to how understudied cell types affect normal and pathophysiological states. The advancement of bioengineered tissue morphogenesis (Fedorchak *et al*. 2020), formation of “assembloids” from region-specific organoids (Xiang *et al*. 2017), and axonal connection of multiple tissues representing the peripheral and central nervous systems (Bowser and Moore 2019) will allow for modeling of distinct neuroanatomical features separated by fibers, akin to how these structures appear *in vivo*. This guided cross-talk of distinct organoids of physiologically relevant tissue will ideally be ever-expanded upon moving forward.

Carrying these ideas forward to different distinct loci within the brain, such as the *substantia nigra* to the *putamen*, will help elucidate complex pathologies that arise from defects of deep brain structures. Better recreating other distinct anatomical features (e.g., the perineurium in the peripheral and the blood-brain barrier in the central nervous system) enables 3D systems to study how drugs and pathogens cross these barriers and affect neural and glial tissue alike. Finally, being able to faithfully record electrophysiological outcomes must move away from planar electrodes found on MEAs and not rely on tissue-disruptive field recordings. While the automation of patch clamping via robotics (Holst *et al*. 2019) expediates a normally time and labor-intensive process (Milligan *et al*. 2009), integrated nanoelectronic electrodes within tissues may very well be the future (Q. Li *et al*. 2019). Developing 3D, integrated electrodes into neural MPSs will allow for long-term, real-time monitoring of spontaneous and inducible activity of cultured tissue (Soscia *et al*. 2020) and provide for more rapid testing of pharmaceuticals within these constructs. Combining the complicated architectures of 3D MPS with appropriate functional aspects to truly create an in vivo mimic will be the key for these systems not only to become major tools for drug screening applications and neurological discoveries but also to replace animal testing completely.

## References

[CR1] Abreu CM, Gama L, Krasemann S, Chesnut M, Odwin-Dacosta S, Hogberg HT, Hartung T, Pamies D (2018). Microglia increase inflammatory responses in iPSC-derived human BrainSpheres. Front Microbiol.

[CR2] Afshar Bakooshli M, Lippmann ES, Mulcahy B, Iyer N, Nguyen CT, Tung K (2019). A 3D culture model of innervated human skeletal muscle enables studies of the adult neuromuscular junction. Elife.

[CR3] Alepee N, Bahinski A, Daneshian M, De Wever B, Fritsche E, Goldberg A (2014). State-of-the-art of 3D cultures (organs-on-a-chip) in safety testing and pathophysiology. ALTEX.

[CR4] Amin ND, Pasca SP (2018). Building models of brain disorders with three-dimensional organoids. Neuron.

[CR5] Anderson WA, Willenberg AR, Bosak AJ, Willenberg BJ, Lambert S (2018). Use of a capillary alginate gel (Capgel) to study the three-dimensional development of sensory nerves reveals the formation of a rudimentary perineurium. J Neurosci Methods.

[CR6] Antill-O’Brien N, Bourke J, O’Connell CD (2019) Layer-by-layer: the case for 3D bioprinting neurons to create patient-specific epilepsy models. Materials (Basel) 12(19). 10.3390/ma12193218 10.3390/ma12193218 PMC6804258 31581436

[CR7] Banks WA (2016). From blood-brain barrier to blood-brain interface: new opportunities for CNS drug delivery. Nat Rev Drug Discov.

[CR8] Barnes JM, Przybyla L, Weaver VM (2017). Tissue mechanics regulate brain development, homeostasis and disease. J Cell Sci.

[CR9] Belle AM, Enright HA, Sales AP, Kulp K, Osburn J, Kuhn EA, Fischer NO, Wheeler EK (2018). Evaluation of in vitro neuronal platforms as surrogates for in vivo whole brain systems. Sci Rep.

[CR10] Bian S, Repic M, Guo Z, Kavirayani A, Burkard T, Bagley JA, Krauditsch C, Knoblich JA (2018). Genetically engineered cerebral organoids model brain tumor formation. Nat Methods.

[CR11] Billiards SS, Haynes RL, Folkerth RD, Borenstein NS, Trachtenberg FL, Rowitch DH, Ligon KL, Volpe JJ, Kinney HC (2008). Myelin abnormalities without oligodendrocyte loss in periventricular leukomalacia. Brain Pathol.

[CR12] Bosi S, Rauti R, Laishram J, Turco A, Lonardoni D, Nieus T, Prato M, Scaini D, Ballerini L (2015). From 2D to 3D: novel nanostructured scaffolds to investigate signalling in reconstructed neuronal networks. Sci Rep.

[CR13] Bowser DA, Moore MJ (2019). Biofabrication of neural microphysiological systems using magnetic spheroid bioprinting. Biofabrication.

[CR14] Bullen CK, Hogberg HT, Bahadirli-Talbott A, Bishai WR, Hartung T, Keuthan C *et al* (2020) Infectability of human BrainSphere neurons suggests neurotropism of SARS-CoV-2. ALTEX. 10.14573/altex.2006111 10.14573/altex.2006111 32591839

[CR15] Campenot RB (1977). Local control of neurite development by nerve growth factor. Proc Natl Acad Sci U S A.

[CR16] Campisi M, Shin Y, Osaki T, Hajal C, Chiono V, Kamm RD (2018). 3D self-organized microvascular model of the human blood-brain barrier with endothelial cells, pericytes and astrocytes. Biomaterials.

[CR17] Centers for Disease Control (2020). New data show significant changes in drug overdose deaths, Atlanta, GA. Retrieved from https://www.cdc.gov/media/releases/2020/p0318-data-show-changes-overdose-deaths.html. Accessed 26 Oct 2020

[CR18] Charles P, Reynolds R, Seilhean D, Rougon G, Aigrot MS, Niezgoda A (2002). Re-expression of PSA-NCAM by demyelinated axons: an inhibitor of remyelination in multiple sclerosis?. Brain.

[CR19] Costa FA, Moreira Neto FL (2015). Satellite glial cells in sensory ganglia: its role in pain. Rev Bras Anestesiol.

[CR20] Dixon TA, Cohen E, Cairns DM, Rodriguez M, Mathews J, Jose RR, Kaplan DL (2018). Bioinspired three-dimensional human neuromuscular junction development in suspended hydrogel arrays. Tissue Eng Part C Methods.

[CR21] Dowden H, Munro J (2019). Trends in clinical success rates and therapeutic focus. Nat Rev Drug Discov.

[CR22] Eldridge CF, Bunge MB, Bunge RP, Wood PM (1987). Differentiation of axon-related Schwann cells in vitro. I. Ascorbic acid regulates basal lamina assembly and myelin formation. J Cell Biol.

[CR23] Enes J, Haburcak M, Sona S, Gerard N, Mitchell AC, Fu W, Birren SJ (2020). Satellite glial cells modulate cholinergic transmission between sympathetic neurons. PLoS One.

[CR24] Environmental Protection Agency (2015). Neurodevelopmental disorders. Retrieved from https://www.epa.gov/sites/production/files/2015-10/documents/ace3_neurodevelopmental.pdf. Accessed 26 Oct 2020

[CR25] Espinosa-Hoyos D, Jagielska A, Homan KA, Du H, Busbee T, Anderson DG (2018). Engineered 3D-printed artificial axons. Sci Rep.

[CR26] Faustino Martins JM, Fischer C, Urzi A, Vidal R, Kunz S, Ruffault PL, Kabuss L, Hube I, Gazzerro E, Birchmeier C, Spuler S, Sauer S, Gouti M (2020). Self-organizing 3D human trunk neuromuscular organoids. Cell Stem Cell.

[CR27] Fedorchak NJ, Iyer N, Ashton RS (2020) Bioengineering tissue morphogenesis and function in human neural organoids. Semin Cell Dev Biol. 10.1016/j.semcdb.2020.05.025 10.1016/j.semcdb.2020.05.025 PMC8755418 32540123

[CR28] Feigin VL, Vos T (2019). Global burden of neurological disorders: from global burden of disease estimates to actions. Neuroepidemiology.

[CR29] Frega M, Tedesco M, Massobrio P, Pesce M, Martinoia S (2014). Network dynamics of 3D engineered neuronal cultures: a new experimental model for in-vitro electrophysiology. Sci Rep.

[CR30] Friston KJ (2011). Functional and effective connectivity: a review. Brain Connect.

[CR31] Garcia-Leon JA, Kumar M, Boon R, Chau D, One J, Wolfs E (2018). SOX10 single transcription factor-based fast and efficient generation of oligodendrocytes from human pluripotent stem cells. Stem Cell Rep.

[CR32] George D, Ahrens P, Lambert S (2018). Satellite glial cells represent a population of developmentally arrested Schwann cells. Glia.

[CR33] George DS, Anderson WA, Sommerhage F, Willenberg AR, Hines RB, Bosak AJ, Willenberg BJ, Lambert S (2019). Bundling of axons through a capillary alginate gel enhances the detection of axonal action potentials using microelectrode arrays. J Tissue Eng Regen Med.

[CR34] Georges PC, Miller WJ, Meaney DF, Sawyer ES, Janmey PA (2006). Matrices with compliance comparable to that of brain tissue select neuronal over glial growth in mixed cortical cultures. Biophys J.

[CR35] Gordon J, Amini S, White MK (2013). General overview of neuronal cell culture. Methods Mol Biol.

[CR36] Grienberger C, Konnerth A (2012). Imaging calcium in neurons. Neuron.

[CR37] Hersh J, Yang SH (2018). Glia-immune interactions post-ischemic stroke and potential therapies. Exp Biol Med (Maywood).

[CR38] Holst GL, Stoy W, Yang B, Kolb I, Kodandaramaiah SB, Li L, Knoblich U, Zeng H, Haider B, Boyden ES, Forest CR (2019). Autonomous patch-clamp robot for functional characterization of neurons in vivo: development and application to mouse visual cortex. J Neurophysiol.

[CR39] Horner PJ, Gage FH (2000). Regenerating the damaged central nervous system. Nature.

[CR40] Hughes CS, Postovit LM, Lajoie GA (2010). Matrigel: a complex protein mixture required for optimal growth of cell culture. Proteomics.

[CR41] Huval RM, Miller OH, Curley JL, Fan Y, Hall BJ, Moore MJ (2015). Microengineered peripheral nerve-on-a-chip for preclinical physiological testing. Lab Chip.

[CR42] Hyvarinen T, Hyysalo A, Kapucu FE, Aarnos L, Vinogradov A, Eglen SJ (2019). Functional characterization of human pluripotent stem cell-derived cortical networks differentiated on laminin-521 substrate: comparison to rat cortical cultures. Sci Rep.

[CR43] Hyysalo A, Ristola M, Makinen ME, Hayrynen S, Nykter M, Narkilahti S (2017). Laminin alpha5 substrates promote survival, network formation and functional development of human pluripotent stem cell-derived neurons in vitro. Stem Cell Res.

[CR44] Johnson BN, Lancaster KZ, Hogue IB, Meng F, Kong YL, Enquist LW, McAlpine MC (2016). 3D printed nervous system on a chip. Lab Chip.

[CR45] Kang SH, Li Y, Fukaya M, Lorenzini I, Cleveland DW, Ostrow LW, Rothstein JD, Bergles DE (2013). Degeneration and impaired regeneration of gray matter oligodendrocytes in amyotrophic lateral sclerosis. Nat Neurosci.

[CR46] Karzbrun E, Kshirsagar A, Cohen SR, Hanna JH, Reiner O (2018). Human brain organoids on a chip reveal the physics of folding. Nat Phys.

[CR47] Kayal C, Moeendarbary E, Shipley RJ, Phillips JB (2019). Mechanical response of neural cells to physiologically relevant stiffness gradients. Adv Healthc Mater.

[CR48] Kim SH, Im SK, Oh SJ, Jeong S, Yoon ES, Lee CJ, Choi N, Hur EM (2017). Anisotropically organized three-dimensional culture platform for reconstruction of a hippocampal neural network. Nat Commun.

[CR49] Kim YS, Jung HM, Yoon BE (2018). Exploring glia to better understand Alzheimer’s disease. Anim Cells Syst (Seoul).

[CR50] Kramer L, Nguyen HT, Jacobs E, McCoy L, Curley JL, Sharma AD, Moore MJ (2020) Modeling chemotherapy-induced peripheral neuropathy using a nerve-on-a-chip microphysiological system. ALTEX. 10.14573/altex.2001181 10.14573/altex.2001181 32388569

[CR51] Krieger TG, Tirier SM, Park J, Jechow K, Eisemann T, Peterziel H, Angel P, Eils R, Conrad C (2020). Modeling glioblastoma invasion using human brain organoids and single-cell transcriptomics. Neuro-Oncology.

[CR52] Lai Y, Cheng K, Kisaalita W (2012). Three dimensional neuronal cell cultures more accurately model voltage gated calcium channel functionality in freshly dissected nerve tissue. PLoS One.

[CR53] Lancaster MA, Renner M, Martin CA, Wenzel D, Bicknell LS, Hurles ME, Homfray T, Penninger JM, Jackson AP, Knoblich JA (2013). Cerebral organoids model human brain development and microcephaly. Nature.

[CR54] Lantoine J, Grevesse T, Villers A, Delhaye G, Mestdagh C, Versaevel M, Mohammed D, Bruyère C, Alaimo L, Lacour SP, Ris L, Gabriele S (2016). Matrix stiffness modulates formation and activity of neuronal networks of controlled architectures. Biomaterials.

[CR55] Lee SY, George JH, Nagel DA, Ye H, Kueberuwa G, Seymour LW (2019). Optogenetic control of iPS cell-derived neurons in 2D and 3D culture systems using channelrhodopsin-2 expression driven by the synapsin-1 and calcium-calmodulin kinase II promoters. J Tissue Eng Regen Med.

[CR56] Leslie DL, Ba DM, Agbese E, Xing X, Liu G (2019) The economic burden of the opioid epidemic on states: the case of Medicaid. Am J Manag Care 25(13 Suppl):S243–S249. Retrieved from https://www.ncbi.nlm.nih.gov/pubmed/31361426. Accessed 26 Oct 2020 31361426

[CR57] Li Q, Nan K, Le Floch P, Lin Z, Sheng H, Blum TS, Liu J (2019). Cyborg organoids: implantation of nanoelectronics via organogenesis for tissue-wide electrophysiology. Nano Lett.

[CR58] Li S, Severino FPU, Ban J, Wang L, Pinato G, Torre V, Chen Y (2018). Improved neuron culture using scaffolds made of three-dimensional PDMS micro-lattices. Biomed Mater.

[CR59] Linkous A, Balamatsias D, Snuderl M, Edwards L, Miyaguchi K, Milner T, Reich B, Cohen-Gould L, Storaska A, Nakayama Y, Schenkein E, Singhania R, Cirigliano S, Magdeldin T, Lin Y, Nanjangud G, Chadalavada K, Pisapia D, Liston C, Fine HA (2019). Modeling patient-derived glioblastoma with cerebral organoids. Cell Rep.

[CR60] Madhavan M, Nevin ZS, Shick HE, Garrison E, Clarkson-Paredes C, Karl M, Clayton BLL, Factor DC, Allan KC, Barbar L, Jain T, Douvaras P, Fossati V, Miller RH, Tesar PJ (2018). Induction of myelinating oligodendrocytes in human cortical spheroids. Nat Methods.

[CR61] Marques S, Zeisel A, Codeluppi S, van Bruggen D, Mendanha Falcao A, Xiao L, Li H, Haring M, Hochgerner H, Romanov RA, Gyllborg D, Munoz-Manchado AB, la Manno G, Lonnerberg P, Floriddia EM, Rezayee F, Ernfors P, Arenas E, Hjerling-Leffler J, Harkany T, Richardson WD, Linnarsson S, Castelo-Branco G (2016). Oligodendrocyte heterogeneity in the mouse juvenile and adult central nervous system. Science.

[CR62] Marton RM, Miura Y, Sloan SA, Li Q, Revah O, Levy RJ (2019). Differentiation and maturation of oligodendrocytes in human three-dimensional neural cultures. Nat Neurosci.

[CR63] Marx U, Akabane T, Andersson TB, Baker E, Beilmann M, Beken S (2020). Biology-inspired microphysiological systems to advance patient benefit and animal welfare in drug development. ALTEX.

[CR64] Marx U, Andersson TB, Bahinski A, Beilmann M, Beken S, Cassee FR (2016). Biology-inspired microphysiological system approaches to solve the prediction dilemma of substance testing. ALTEX.

[CR65] Milligan CJ, Li J, Sukumar P, Majeed Y, Dallas ML, English A, Emery P, Porter KE, Smith AM, McFadzean I, Beccano-Kelly D, Bahnasi Y, Cheong A, Naylor J, Zeng F, Liu X, Gamper N, Jiang LH, Pearson HA, Peers C, Robertson B, Beech DJ (2009). Robotic multiwell planar patch-clamp for native and primary mammalian cells. Nat Protoc.

[CR66] Mitani A, Komiyama T (2018). Real-time processing of two-photon calcium imaging data including lateral motion artifact correction. Front Neuroinform.

[CR67] Mohs RC, Greig NH (2017). Drug discovery and development: role of basic biological research. Alzheimers Dement (N Y).

[CR68] Monje PV (2020) Schwann cell cultures: biology, technology and therapeutics. Cells 9(8). 10.3390/cells9081848 10.3390/cells9081848 PMC7465416 32781699

[CR69] Morante-Redolat JM, Porlan E (2019). Neural stem cell regulation by adhesion molecules within the subependymal niche. Front Cell Dev Biol.

[CR70] Moutaux E, Charlot B, Genoux A, Saudou F, Cazorla M (2018). An integrated microfluidic/microelectrode array for the study of activity-dependent intracellular dynamics in neuronal networks. Lab Chip.

[CR71] Musto M, Rauti R, Rodrigues AF, Bonechi E, Ballerini C, Kostarelos K, Ballerini L (2019). 3D organotypic spinal cultures: exploring neuron and neuroglia responses upon prolonged exposure to graphene oxide. Front Syst Neurosci.

[CR72] Najjar S, Pearlman DM (2015). Neuroinflammation and white matter pathology in schizophrenia: systematic review. Schizophr Res.

[CR73] Nayernia Z, Turchi L, Cosset E, Peterson H, Dutoit V, Dietrich PY, Tirefort D, Chneiweiss H, Lobrinus JA, Krause KH, Virolle T, Preynat-Seauve O (2013). The relationship between brain tumor cell invasion of engineered neural tissues and in vivo features of glioblastoma. Biomaterials.

[CR74] Nguyen H, McCoy L, Willey H, Sharma AD, Curley L, Moore M (2019). Nerve-on-a-chip platform for assessing chemotherapy-induced peripheral neuropathy. J Pharmacol Toxicol Methods.

[CR75] Nikolakopoulou P, Rauti R, Voulgaris D, Shlomy I, Maoz BM, Herland A (2020) Recent progress in translational engineered in vitro models of the central nervous system. Brain. 10.1093/brain/awaa268 10.1093/brain/awaa268 PMC7719033 33020798

[CR76] Nirwane A, Yao Y (2018). Laminins and their receptors in the CNS. Biol Rev Camb Philos Soc.

[CR77] Noseworthy JH, Lucchinetti C, Rodriguez M, Weinshenker BG (2000). Multiple sclerosis. N Engl J Med.

[CR78] Nzou G, Wicks RT, Wicks EE, Seale SA, Sane CH, Chen A, Murphy SV, Jackson JD, Atala AJ (2018). Human cortex spheroid with a functional blood brain barrier for high-throughput neurotoxicity screening and disease modeling. Sci Rep.

[CR79] Obien ME, Deligkaris K, Bullmann T, Bakkum DJ, Frey U (2014). Revealing neuronal function through microelectrode array recordings. Front Neurosci.

[CR80] Ogawa J, Pao GM, Shokhirev MN, Verma IM (2018). Glioblastoma model using human cerebral organoids. Cell Rep.

[CR81] Omar A, Marwaha K, Bollu PC (2020) Physiology, neuromuscular junction. In StatPearls. Treasure Island (FL) 29261907

[CR82] Osaki T, Uzel SGM, Kamm RD (2020). On-chip 3D neuromuscular model for drug screening and precision medicine in neuromuscular disease. Nat Protoc.

[CR83] Pamies D, Barreras P, Block K, Makri G, Kumar A, Wiersma D, … Hogberg HT (2017). A human brain microphysiological system derived from induced pluripotent stem cells to study neurological diseases and toxicity. ALTEX 34(3):362–376. 10.14573/altex.1609122 10.14573/altex.1609122 PMC6047513 27883356

[CR84] Pamies D, Block K, Lau P, Gribaldo L, Pardo CA, Barreras P, Smirnova L, Wiersma D, Zhao L, Harris G, Hartung T, Hogberg HT (2018). Rotenone exerts developmental neurotoxicity in a human brain spheroid model. Toxicol Appl Pharmacol.

[CR85] Pellegrini L, Bonfio C, Chadwick J, Begum F, Skehel M, Lancaster MA (2020). Human CNS barrier-forming organoids with cerebrospinal fluid production. Science..

[CR86] Pistollato, F., Ohayon, E. L., Lam, A., Langley, G. R., Novak, T. J., Pamies, D., … Chandrasekera, P. C. (2016). Alzheimer disease research in the 21st century: past and current failures, new perspectives and funding priorities. Oncotarget, 7(26), 38999-39016. doi:10.18632/oncotarget.9175 10.18632/oncotarget.9175 PMC5129909 27229915

[CR87] Plummer S, Wallace S, Ball G, Lloyd R, Schiapparelli P, Quinones-Hinojosa A (2019). A Human iPSC-derived 3D platform using primary brain cancer cells to study drug development and personalized medicine. Sci Rep.

[CR88] Pollard K, Sharma AD, Moore M (2019). Neural microphysiological systems for in vitro modeling of peripheral nervous system disorders. Bioelectron Med.

[CR89] Previtera ML, Langhammer CG, Firestein BL (2010). Effects of substrate stiffness and cell density on primary hippocampal cultures. J Biosci Bioeng.

[CR90] Qian X, Nguyen HN, Song MM, Hadiono C, Ogden SC, Hammack C, Yao B, Hamersky GR, Jacob F, Zhong C, Yoon KJ, Jeang W, Lin L, Li Y, Thakor J, Berg DA, Zhang C, Kang E, Chickering M, Nauen D, Ho CY, Wen Z, Christian KM, Shi PY, Maher BJ, Wu H, Jin P, Tang H, Song H, Ming GL (2016). Brain-region-specific organoids using mini-bioreactors for modeling ZIKV exposure. Cell.

[CR91] Renault R, Sukenik N, Descroix S, Malaquin L, Viovy JL, Peyrin JM, Bottani S, Monceau P, Moses E, Vignes M (2015). Combining microfluidics, optogenetics and calcium imaging to study neuronal communication in vitro. PLoS One.

[CR92] Rouwkema J, Koopman B, Blitterswijk C, Dhert W, Malda J (2010). Supply of nutrients to cells in engineered tissues. Biotechnol Genet Eng Rev.

[CR93] Sakai K, Shimba K, Kotani K, Jimbo Y (2017). A co-culture microtunnel technique demonstrating a significant contribution of unmyelinated Schwann cells to the acceleration of axonal conduction in Schwann cell-regulated peripheral nerve development. Integr Biol (Camb).

[CR94] Santhanam N, Kumanchik L, Guo X, Sommerhage F, Cai Y, Jackson M, Martin C, Saad G, McAleer CW, Wang Y, Lavado A, Long CJ, Hickman JJ (2018). Stem cell derived phenotypic human neuromuscular junction model for dose response evaluation of therapeutics. Biomaterials.

[CR95] Savtchouk I, Carriero G, Volterra A (2018). Studying axon-astrocyte functional interactions by 3D two-photon Ca(2+) imaging: a practical guide to experiments and “big data” analysis. Front Cell Neurosci.

[CR96] Schafer DP, Lehrman EK, Stevens B (2013). The “quad-partite” synapse: microglia-synapse interactions in the developing and mature CNS. Glia.

[CR97] Schmidt BZ, Lehmann M, Gutbier S, Nembo E, Noel S, Smirnova L, … Dinnyes A (2017) In vitro acute and developmental neurotoxicity screening: an overview of cellular platforms and high-throughput technical possibilities. Arch Toxicol, 91(1):1-33. doi:10.1007/s00204-016-1805-9 10.1007/s00204-016-1805-9 27492622

[CR98] Sharma AD, McCoy L, Jacobs E, Willey H, Behn JQ, Nguyen H, Bolon B, Curley JL, Moore MJ (2019). Engineering a 3D functional human peripheral nerve in vitro using the nerve-on-a-chip platform. Sci Rep.

[CR99] Shin Y, Choi SH, Kim E, Bylykbashi E, Kim JA, Chung S, Kim DY, Kamm RD, Tanzi RE (2019). Blood-brain barrier dysfunction in a 3D in vitro model of Alzheimer’s disease. Adv Sci (Weinh).

[CR100] Simao D, Terrasso AP, Teixeira AP, Brito C, Sonnewald U, Alves PM (2016). Functional metabolic interactions of human neuron-astrocyte 3D in vitro networks. Sci Rep.

[CR101] Sloan SA, Darmanis S, Huber N, Khan TA, Birey F, Caneda C, … Pasca SP (2017) Human astrocyte maturation captured in 3D cerebral cortical spheroids derived from pluripotent stem cells. Neuron, 95(4):779-790 e776. doi:10.1016/j.neuron.2017.07.035 10.1016/j.neuron.2017.07.035 PMC5890820 28817799

[CR102] Smirnova L, Hogberg HT, Leist M, Hartung T (2014). Developmental neurotoxicity - challenges in the 21st century and in vitro opportunities. ALTEX.

[CR103] Soscia DA, Lam D, Tooker AC, Enright HA, Triplett M, Karande P, Peters SKG, Sales AP, Wheeler EK, Fischer NO (2020). A flexible 3-dimensional microelectrode array for in vitro brain models. Lab Chip.

[CR104] Tanaka A, Fujii Y, Kasai N, Okajima T, Nakashima H (2018). Regulation of neuritogenesis in hippocampal neurons using stiffness of extracellular microenvironment. PLoS One.

[CR105] Tremblay ME, Cookson MR, Civiero L (2019). Glial phagocytic clearance in Parkinson’s disease. Mol Neurodegener.

[CR106] Trujillo CA, Gao R, Negraes PD, Gu J, Buchanan J, Preissl S, Wang A, Wu W, Haddad GG, Chaim IA, Domissy A, Vandenberghe M, Devor A, Yeo GW, Voytek B, Muotri AR (2019). Complex oscillatory waves emerging from cortical organoids model early human brain network development. Cell Stem Cell.

[CR107] Tuft BW, Zhang L, Xu L, Hangartner A, Leigh B, Hansen MR, Guymon CA (2014). Material stiffness effects on neurite alignment to photopolymerized micropatterns. Biomacromolecules.

[CR108] Ulloa Severino FP, Ban J, Song Q, Tang M, Bianconi G, Cheng G, Torre V (2016). The role of dimensionality in neuronal network dynamics. Sci Rep.

[CR109] Vereyken EJ, Fluitsma DM, Bolijn MJ, Dijkstra CD, Teunissen CE (2009). An in vitro model for de- and remyelination using lysophosphatidyl choline in rodent whole brain spheroid cultures. Glia.

[CR110] Villabona-Rueda A, Erice C, Pardo CA, Stins MF (2019). The evolving concept of the blood brain barrier (BBB): from a single static barrier to a heterogeneous and dynamic relay center. Front Cell Neurosci.

[CR111] Walsh P, Truong V, Hill C, Stoflet ND, Baden J, Low WC, Keirstead SA, Dutton JR, Parr AM (2017). Defined culture conditions accelerate small-molecule-assisted neural induction for the production of neural progenitors from human-induced pluripotent stem cells. Cell Transplant.

[CR112] Wen YQ, Gao X, Wang A, Yang Y, Liu S, Yu Z, Song GB, Zhao HC (2018). Substrate stiffness affects neural network activity in an extracellular matrix proteins dependent manner. Colloids Surf B: Biointerfaces.

[CR113] Xiang Y, Tanaka Y, Patterson B, Kang YJ, Govindaiah G, Roselaar N, Cakir B, Kim KY, Lombroso AP, Hwang SM, Zhong M, Stanley EG, Elefanty AG, Naegele JR, Lee SH, Weissman SM, Park IH (2017). Fusion of regionally specified hPSC-derived organoids models human brain development and interneuron migration. Cell Stem Cell.

[CR114] Xu H, Li Z, Yu Y, Sizdahkhani S, Ho WS, Yin F, Wang L, Zhu G, Zhang M, Jiang L, Zhuang Z, Qin J (2016). A dynamic in vivo-like organotypic blood-brain barrier model to probe metastatic brain tumors. Sci Rep.

[CR115] Yang W, Miller JE, Carrillo-Reid L, Pnevmatikakis E, Paninski L, Yuste R, Peterka DS (2016). Simultaneous multi-plane imaging of neural circuits. Neuron.

[CR116] Zhan J, Mann T, Joost S, Behrangi N, Frank M, Kipp M (2020) The cuprizone model: dos and do nots. Cells 9(4). 10.3390/cells9040843 10.3390/cells9040843 PMC7226799 32244377

[CR117] Zhang QY, Zhang YY, Xie J, Li CX, Chen WY, Liu BL, Wu XA, Li SN, Huo B, Jiang LH, Zhao HC (2014). Stiff substrates enhance cultured neuronal network activity. Sci Rep.

[CR118] Zhong X, Harris G, Smirnova L, Zufferey V, Sa R, Baldino Russo F (2020). Antidepressant paroxetine exerts developmental neurotoxicity in an iPSC-derived 3D human brain model. Front Cell Neurosci.

[CR119] Zonouzi M, Berger D, Jokhi V, Kedaigle A, Lichtman J, Arlotta P (2019). Individual oligodendrocytes show bias for inhibitory axons in the neocortex. Cell Rep.

